# Revealing the Influence of Microparticles on Geopolymers’ Synthesis and Porosity

**DOI:** 10.3390/ma13143211

**Published:** 2020-07-18

**Authors:** Dumitru Doru Burduhos Nergis, Petrica Vizureanu, Ioan Ardelean, Andrei Victor Sandu, Ofelia Cornelia Corbu, Ecaterina Matei

**Affiliations:** 1 Faculty of Materials Science and Engineering, Gheorghe Asachi Technical University of Iași, 700050 Iasi, Romania; bunduc.doru@yahoo.com; 2 Department of Physics and Chemistry, Technical University of Cluj Napoca, 400114 Cluj-Napoca, Romania; ioan.ardelean@phys.utcluj.ro; 3 Faculty of Civil Engineering, Technical University of Cluj-Napoca, 400114 Cluj-Napoca, Romania; ofelia.corbu@staff.utcluj.ro; 4 Faculty of Materials Science and Engineering, Politehnica University of Bucharest, 060042 Bucharest, Romania; ecaterina.matei@ecomet.pub.ro

**Keywords:** coal ash-based geopolymers, NMR relaxation, industrial waste, geopolymer, chemical structure

## Abstract

Geopolymers are zeolites like structures based on hydrated aluminosilicates units of SiO_4_ and AlO_4_. These units, known as poly(sialate), poly(sialate)-siloxo or poly(sialate)-disiloxo are chemically balanced by the group I cations of K^+^, Li^+^, or Na^+^. Simultaneously, the chemical reaction of formation, known as geopolymerization, governs the orientation of the unit, generating mesoporous structures. Multiple methods can be used for pore structure and porosity characterization. Among them, nuclear magnetic resonance (NMR) relaxometry allows the detection of the porous structure in a completely nonperturbative manner. NMR relaxometry may be used to monitor the relaxation of protons belonging to the liquid molecules confined inside the porous structure and, thus, to get access to the pore size distribution. This monitoring can take place even during the polymerization process. The present study implements transverse relaxation measurements to monitor the influence introduced by the curing time on the residual liquid phase of geopolymers prepared with two different types of reinforcing particles. According to our results, the obtained geopolymers contain three types of pores formed by the arrangement of the OH^−^ and Si groups (Si-OH), Si-O-Si groups, Si-O-Al groups, and Si-O rings. After 48 days, the samples cured for 8 h show a high percentage of all three types of pores, however, by increasing the curing time and the percentage of reinforcing particle, the percent of pores decrease, especially, the gel pores.

## 1. Introduction

After mixing a material rich in aluminum and silicon oxides with a strongly alkaline solution a binder is formed which through the geopolymerization chemical reaction passes into a tetragonal Si-O-Al structure, resulting in an inorganic material called geopolymer [1,2,3]. Due to their physical [4], chemical, and mechanical properties, geopolymers present high interest in many industrial applications. Initially, these materials were developed as high fire resistance materials ideal for civil engineering [5,6], but later multiple fields, such as automotive [7], ceramic [8], metallurgical [9], aerospace [10], etc., started using geopolymers as substitutes for conventional oxide materials or polymers. Moreover, their microstructure contains several unreacted particles that continuously react with the gel remaining in the micropores [11]. As a result, some defects (cracks) can be repaired by the self-healing mechanism [12,13]. This self-healing characteristic positively influences the time behavior (durability) of geopolymers. 

However, both the properties and the quality of the geopolymers are strongly affected by multiple parameters [14,15] specific to the mixed components, but also by the obtaining process. According to previous studies, the geopolymers depend on the chemical composition of the raw material [16], humidity [17], particles’ dimensions [18], liquid to solid ratio [19], type of activator [20], curing time [21], temperature [22], and reinforcing particles characteristics [3,23], if used. Moreover, the pore size and distribution can negatively influence, in particular, the main mechanical properties of the geopolymers, as the structure compactness decreases allowing the water or acidic substances to penetrate the sample. According to another study [24], the pores type and size depend, mainly, on the raw material calcium content because this contributes to the formation of the porous phases such as calcium alumino-silicate hydrate (C-A-S-H) and sodium alumino-silicate hydrate (N-A-S-H). Also, it was found that the total porosity and pore size distribution are the most important factors affecting the compressive strength of geopolymers [25,26].

Proton nuclear magnetic resonance (NMR) relaxometry is a valuable technique that can be used to extract information about the pore size distribution of porous materials. The technique relies on the proportionality between the pore size and the relaxation time (transverse or longitudinal) of protons belonging to the liquid molecules confined inside pores [27,28,29]. Thus, from the relaxation time distribution, it is possible to extract the pore size distribution. Note, however, that the proportionality between the pore size and relaxation time is valid only if one neglects the bulk relaxation rate of the confined molecules. Moreover, in the case of porous media with magnetic impurities, it is necessary to reduce diffusion effects on transverse relaxation measurements [26,27]. A valuable approach to reduce diffusion effects on transverse relaxation measurements is to implement the well-known Carr Purcell Meiboom Gill (CPMG) technique [30] in combination with a lowfield NMR instrument.

The geopolymers are porous materials and the effect of high porosity results in low mechanical properties. Consequently, in order to improve certain characteristics, such as compressive or bending strength [3] of geopolymers, different types of aggregates can be introduced into the solid component. In the present work, two types of aggregates will be considered: powdered glass (PG) and sand (S). They will be gradually introduced in the coal-ash based geopolymers and their effect on the relative pore size distribution and chemical structure will be investigated using proton NMR relaxometry and Fourier Transform Infrared Spectroscopy (FTIR) spectroscopy.

## 2. Materials and Methods

### 2.1. Materials

Coal-ash is a secondary product resulted from coal combustion in the burning room of the city’s power plants. The chemical composition analyzed by X-ray fluorescence (XRF) using an XRF S8 Tiger equipment (Bruker GmbH, Karlsruhe, Germany) shows high silica and alumina content being suitable for geopolymers synthesis. However, because different dumps present various chemical compositions the obtaining process must be particularly designed, to obtain a final material with high performances. In this study, the coal-ash (FA) from Holboca CET II (S.C. C.E.T. Iasi S.A., Iasi, Romania) power plant, with a bulk density of 2.16 ± 0.01 g/cm^3^, has been used as raw material. 

Other parameters related to the coal-ash which influence the final characteristics of geopolymers are humidity and particle dimension. Therefore, prior mixing with the activating solution the raw materials have been dried at 110 ± 5 °C until the humidity was removed (considered when there is no weight loss for 30 min. of heating). After the drying stage, the powder has been sieved, according to SR EN 933-1:2012, to remove the large impurities. 

The PG was obtained by crushing and milling glass bottles and containers from the food industry. Further, the PG was sifted and the particles that passed the 1250 mesh sieve, i.e., particles finer than 10 µm, were used for the geopolymers. 

The natural aggregates, i.e., sand, used in this study contains particles dimensions in the range of 0 to 4 mm [3], and a bulk density of 1.41 ± 0.01 g/cm^3^. These particles have a high content of silica and ferrous oxides [31].

The coal-ash collected can be activated with an alkaline solution of sodium silicate (SS) and 10 M sodium hydroxide (NaOH) in the ration of SS to NaOH of 1.5, according to previous studies [3,32]. A commercially purchased high purity SS solution (SO06401000 Sodium Silicate, Scharlab S.L., Barcelona, Spain) with a density of 1.37 g/cm^3^ and a lower pH than 11.5 was used in this study. The NaOH solution was prepared at a 10-molar concentration by dissolving the commercially purchased high purity (99%) NaOH flakes (M-1500 Sodium Hydroxide, Elemental S.R.L., Bihor, Romania) in distilled water for 24 h before use.

### 2.2. Sample Preparation

**Figure 1:**
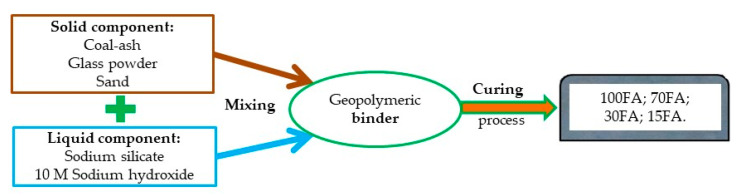
Process flow diagram of sample obtaining.

To control the porosity of the sample, two types of reinforcing particles have been introduced in the geopolymers matrix. To evaluate the effects of curing time, the type and the quantity of reinforcing particles, on the relative distribution of pores, four different compositions, for the solid component, were considered: 100% coal-ash (sample 100 FA); 70% coal-ash and 30% glass powder (sample 70 FA), 30% coal-ash and 70% sand (sample 30 FA) 15% coal-ash, 15% glass powder, and 70% sand (sample 15 FA), respectively, which were subjected to a curing process at 70 °C for three different periods: 8, 16, and 24 h (Figure 1).

The solid component was mixed with the activating solution, in a solid to liquid ratio of 1, using a variable speed mixer for 10 minutes until a homogeneous binder was obtained. In the case of samples with multi constituents in the solid component (70 FA, 30 FA, and 15 FA), these must be mixed in a dry state before introducing the activation solution. Activating the geopolymer with a multi-component solution involves mixing these before introducing the solid component. After mixing, the binder was poured in cylindrical shape molds and subjected to vibrations to reduce the air bubbles caught inside.

Therefore, the following materials and technological parameters were used in this study:

- Coal-ash particles lower than 80 µm;
- Glass powder particles lower than 10 µm;
- Sand particles lower than 4 mm;
- Raw material relative humidity close to 0%;
- Raw material percentage of silicon and aluminum oxides higher than 75%;
- Curing temperature 70 °C;
- Curing time of 8, 16 or 24 h;
- Solid to liquid ratio of 1; and
- Sodium silicate to 10 M sodium hydroxide ratio of 1.5.

### 2.3. Methods

The microstructural analysis was performed by a Scanning Electron Microscope with field emission type FEI Quanta FEG 450 (FEI Company, Washington, DC, USA.) and a Polarized Light Microscope type Axio Imager A1m (Carl Zeiss AG, Oberkochen, Germany). The bulk density was determined through a Densimètre Le Chatelier, and the values presented are the mean value of three determinations. 

#### 2.3.1. NMR Relaxometry

Transverse relaxation measurements of the proton spins confined inside geopolymers were performed using the CPMG technique [28]. Recording of the CPMG echo trains was performed using a low field NMR instrument, operating at 20 MHz proton resonance frequency (Minispec MQ20, Bruker Optics, Bremen, Germany). The echo time used in our investigations was 0.1 ms, which allowed neglecting of the diffusion effects on echo train attenuation. The NMR measurements were performed first on samples maintained in fresh air at room temperature conditions (21–23 °C; 40–48% air humidity) for 48 days to highlight the residual activator in pores. Then all the samples were immersed in water for 7 days and measured again in order to evaluate water absorption and the relative size distribution of all pores in the structure. The relaxation time distribution was obtained from the CPMG echo trains using a numerical Laplace transform [33]. Provided that one can neglect the bulk contribution to the relaxation rate and the interaction of confined molecules with the surface of the investigated samples is identical, then the relaxation time distribution mimics the pore size distribution.

#### 2.3.2. Fourier Transform Infrared Spectroscopy 

FTIR is a non-destructive technique for analyzing the chemical structure of a material. This consists of obtaining an infrared light absorption spectrum at different wavelengths of a beam. The analysis was performed using a Bruker Hyperion 1000 FTIR spectrometer (Bruker Optics, Bremen, Germany), coupled with a microscope (Bruker Optics, Bremen, Germany), equipped with a 15× lens. Due to the use of the microscope, the samples didn’t need to be embedded in KBr pellets. Therefore, the analysis was carried out directly on polished samples in a range of wave numbers between 4000 cm^−1^ and 600 cm^−1^ with a resolution of 4 cm^−1^ at a scan frequency of 10 kHz through a 6 mm diameter aperture and 64 scans for each surface. The absorbance spectrum of the samples show multiple peaks included in the vibration bands of the chemical bonds in the present groups. The spectra were analyzed using OPUS 65 Bruker (Bruker Optics, Bremen, Germany) software to study, in particular, the groups formed between Si, Al, H, and O.

## 3. Results and Discussion

### Raw Materials Characterization

**Figure 2:**
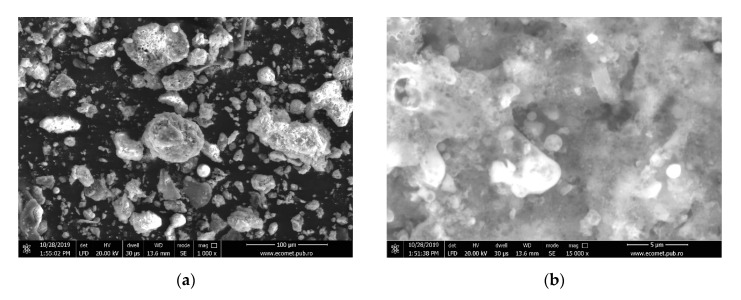
Indigenous coal-ash morphology: (**a**) 1000× SEM micrography; (**b**) 15,000× SEM micrography.

According to the chemical composition (Table 1) the coal-ash powder used as raw material belongs to class F fly ashes (ASTM C618-92a). Figure 2 show the coal-ash particle morphology after sifting. As can be seen, at a magnification ratio of 1,000× times the coal-ash particles, are porous bodies with different sizes and shapes (Figure 2a), while at a magnification ratio of 15,000×, particles with round shape, i.e., fly ash, accumulated in a porous matrix can be observed (Figure 2b).

**Figure 3:**
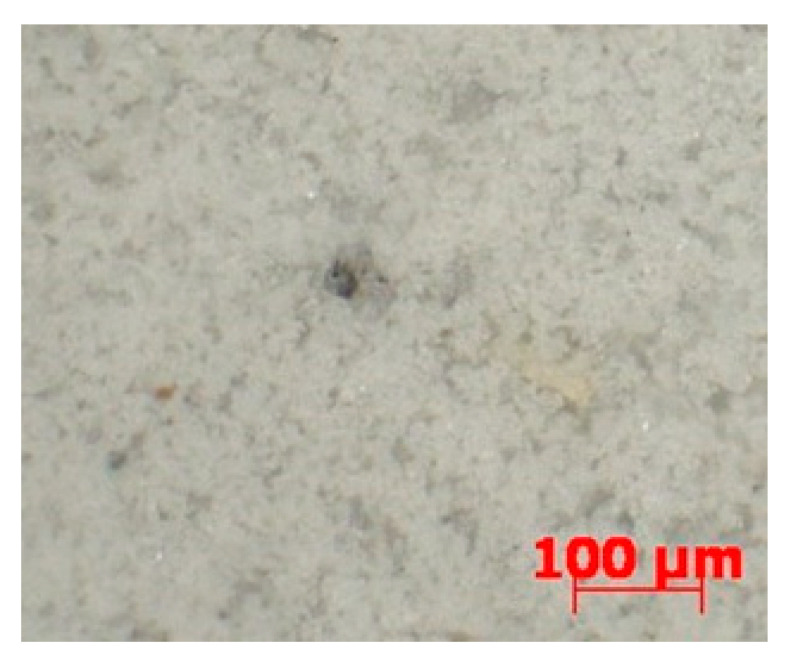
PG morphology.

The PG (Figure 3) used, with a bulk density of 2.52 ± 0.01 g/cm^3^, has low aluminum and iron oxides contents (Table 2) and it reacts in alkaline environments [34]. Consequently, the introduction of PG into geopolymers contributes positively to the geopolymerization reaction, due to its pozzolanic activity [35].

**Figure 4:**
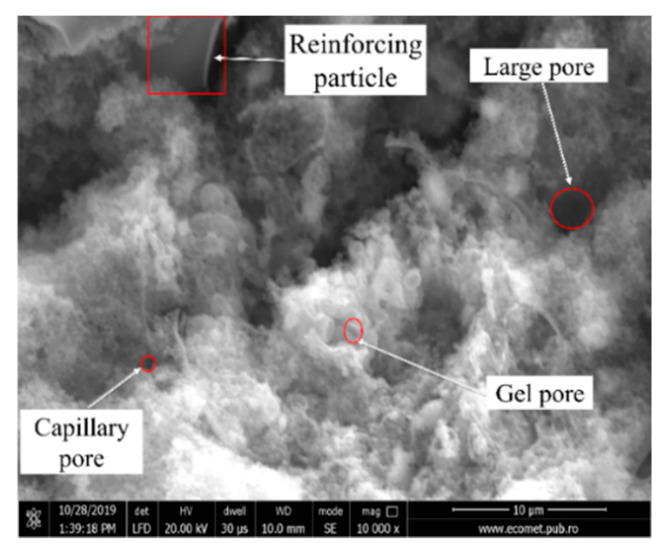
Coal-ash based geopolymers morphology.

From a structural point of view, the geopolymers contain unreacted raw material due to several factors, such as improper mixing, too low a liquid to solid ratio, etc. This can be observed in the form of surfaces covered with spheres (ash particles) in SEM micrographs (Figure 4). However, there is no exact method to quantitatively evaluate the unreacted ash from a specific sample. Due to their spherical structure, the coal-ash particles which do not react before the setting time ending, influence significantly the porosity of the geopolymers.

**Figure 5:**
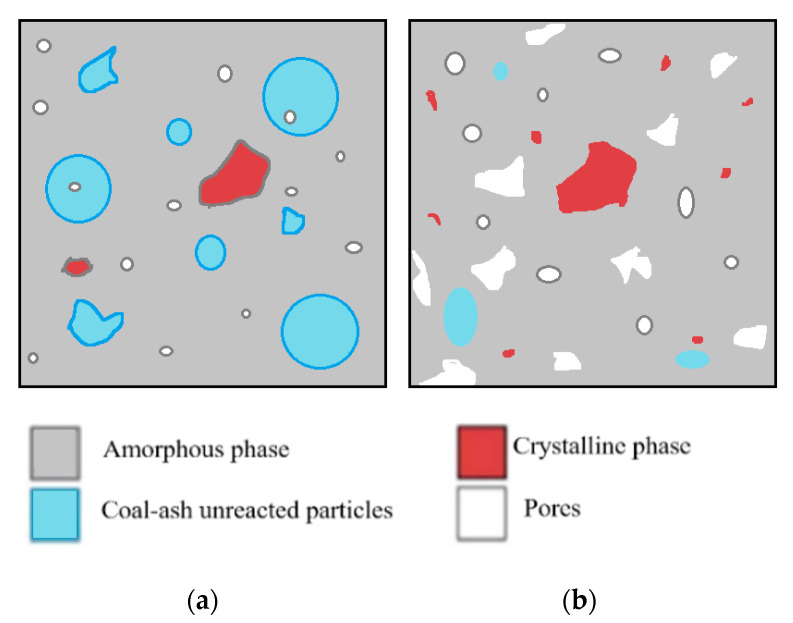
Schematic representation of geopolymers morphology: (**a**) after setting time ending and (**b**) after reacting [36].

Following the formation of the solid structure (Figure 5a), the regions inside the samples containing ash particles and activation solution will react in time creating pores of different sizes. Also, the pores formation or their increase in size could be affected by the curing time increase, because the conversion of the amorphous phase into the crystalline phase occurs during the curing stage (Figure 5b).

**Figure 6:**
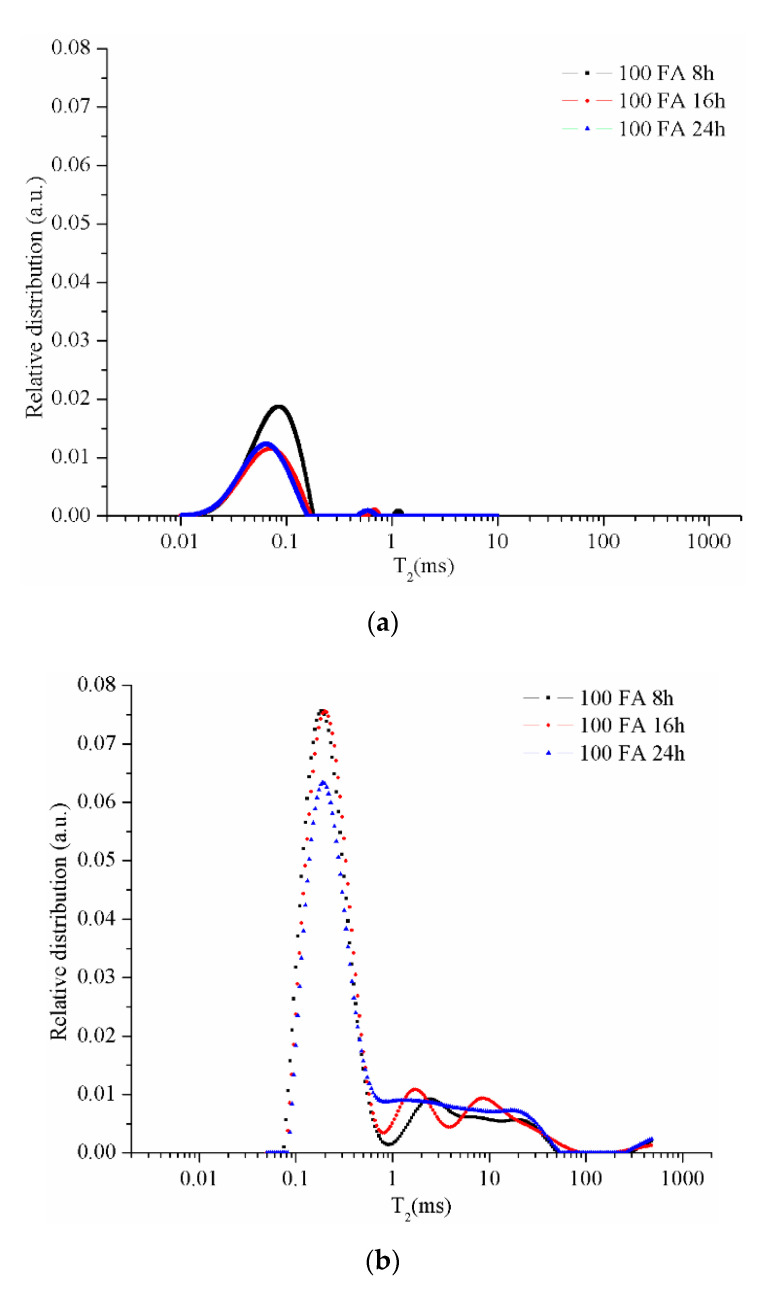
Relative pore size distribution in sample 100 FA dried for 8, 16, or 24 h: (**a**) after 48 days of activation and (**b**) after 7 days of immersion in water.

**Figure 7:**
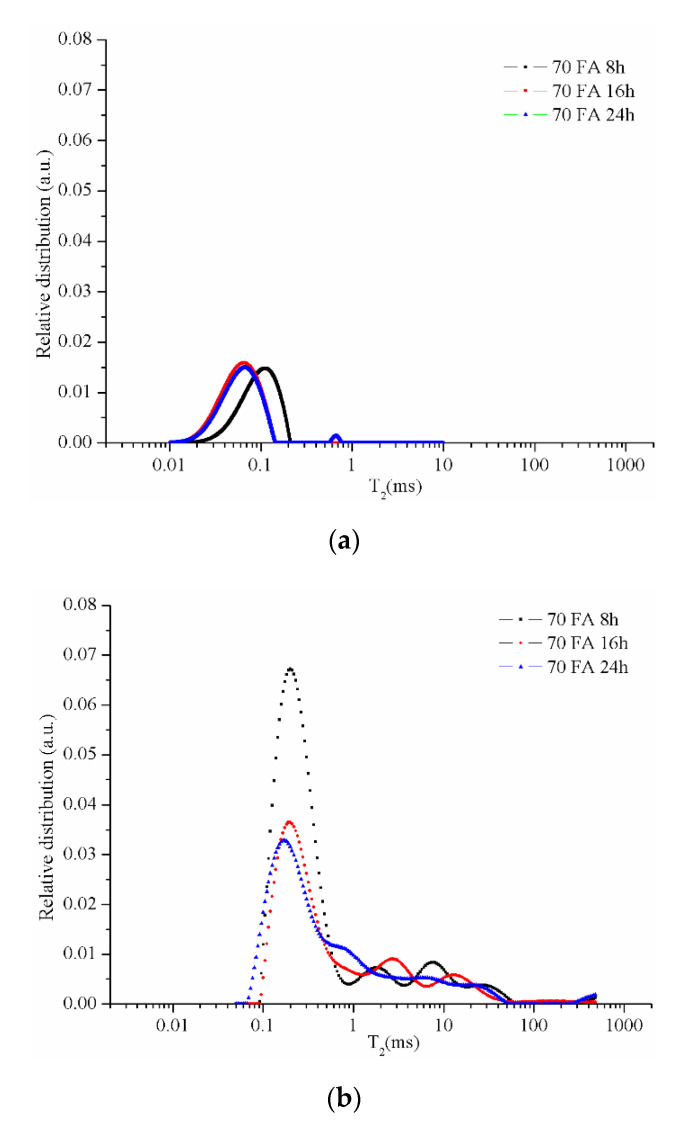
Relative pore size distribution in sample 70 FA dried for 8, 16, or 24 h: (**a**) after 48 days of activation and (**b**) after 7 days of immersion in water.

**Figure 8:**
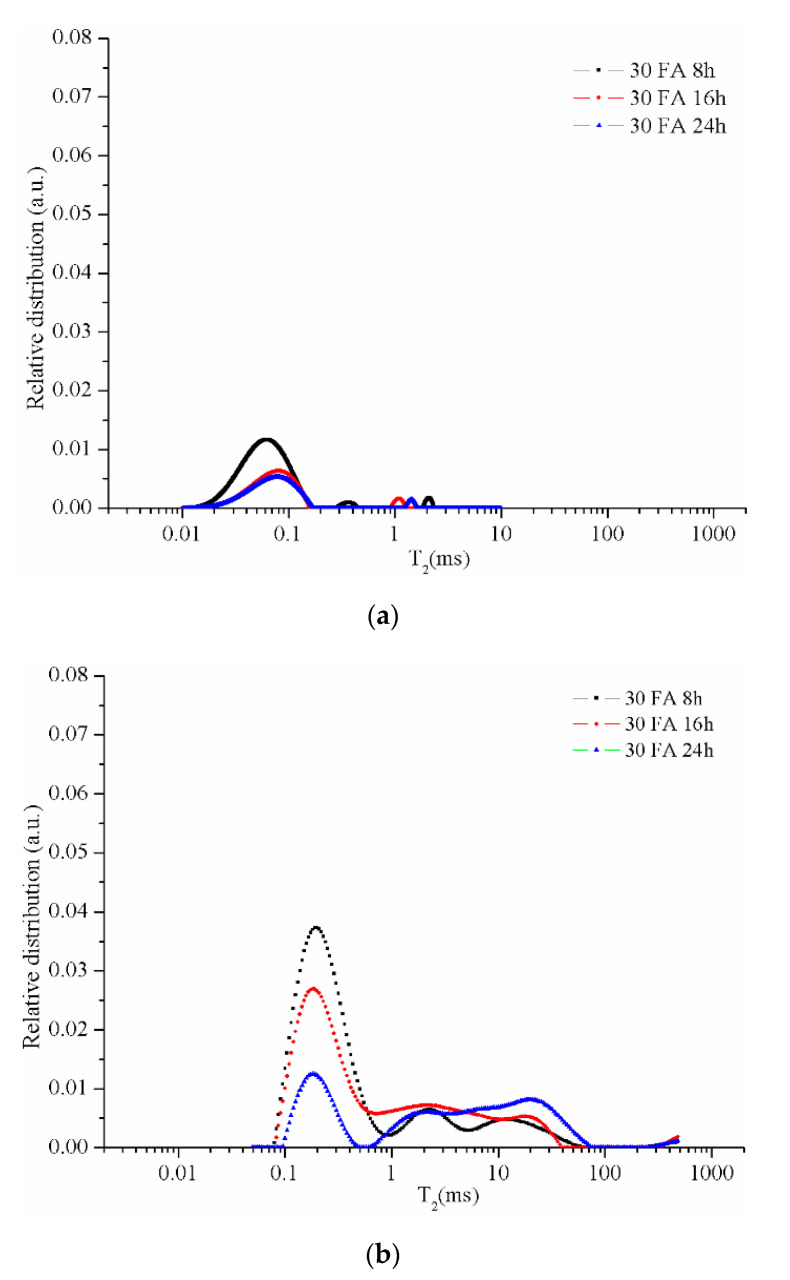
Relative pore size distribution in sample 30 FA dried for 8, 16, or 24 h: (**a**) after 48 days of activation and (**b**) after 7 days of immersion in water.

**Figure 9:**
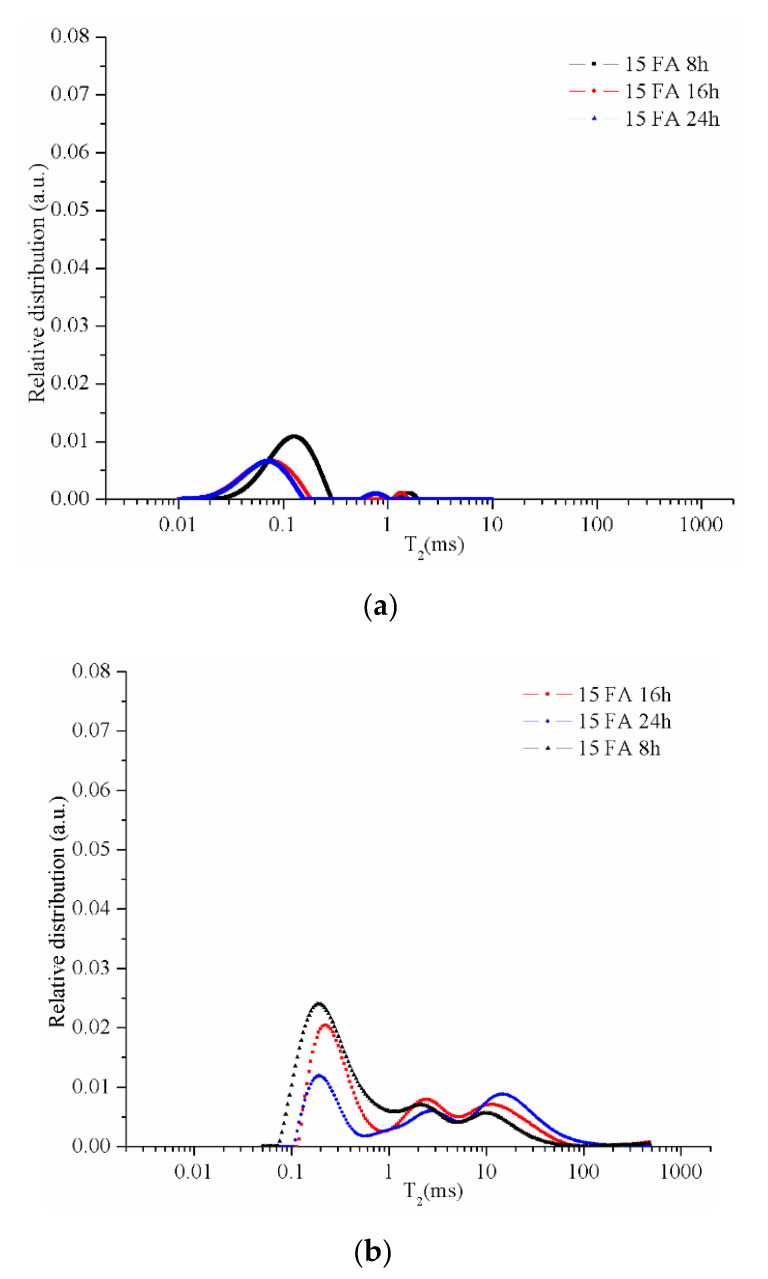
Relative pore size distribution in sample 15 FA dried for 8, 16, or 24 h: (**a**) after 48 days of activation and (**b**) after 7 days of immersion in water.

The CPMG series collected from the NMR measurements were used to obtain the relaxation time distributions (T_2_) as presented in the following figures. The resulting peaks were analyzed by a comparison between different types of samples. The graphs specific to the samples maintained in the room conditions (21–23 °C; 40–48 % air humidity) for 48 days (Figure 6a, Figure 7a, Figure 8a and Figure 9a) reveal two peaks. The first peak can be assigned to the liquid protons (residual activator solution) resulting from the geopolymerization process in the partially filled gel pores. The second peak can be attributed to the water absorbed inside the capillary pores from the atmosphere or can be an artifact of the numerical Laplace inversion [28]. It is known that numerical inverse Laplace is ill-conditioned and may lead to spurious peaks when applied to noisy data. Comparing the data on the samples maintained at room conditions with those of the samples immersed in water for 7 days, one can observe a significant increase of the peak area which is proportional to the number of protons confined inside the pores. It is also observed that different types of pores cannot be graphically separated due to the rapid exchange of water molecules from one type of pore to another.

The graph of the sample 100 FA (Figure 6b) dried for 8 h shows several peaks on the curve (black dots curve), the first peak between ≈0.1 ms and 1 ms corresponds to the liquid in the gel-type pores (<50 nm), the second peak between ≈1 ms and 7.5 ms corresponds to the capillary pores (50–600 nm), and the third peak >7.5 ms corresponds to the liquid in the voids (pores larger than 600 nm) or the cracks results from the crystalline phase growth [32,37]. As the drying time increases, the gel remaining on the surface of the unreacted or partially dissolved coal-ash particles continues to activate, resulting in the ash spheres opening, therefore, pores volume increases. At the same time, the density of the sample decreases as a result of the structure permeability increase due to the remaining water elimination from the small pores.

As can be seen in Figure 6b, the effect of drying time increasing, from 8 to 16 h, on the gel pore size distribution is minimum, but in the case of 24 h dried samples the amount of liquid detected is much smaller. This phenomenon can be explained by the decrease in the number of gel pores following the reaction between the activator and the unreacted ash particles, i.e., pore growth. Therefore, the increase of the structure dehydration degree as a result of the drying time increase produces a decrease in the total volume occupied by gel-type pores.

When replacing 30% of the FA with glass particles smaller than 10 µm in diameter, a significant decrease in gel pores occurs, mostly because the sample volume is filled with compact particles. As the drying time increases, the number of pores specific to the first peak decreases significantly, but the characteristic curve of the sample maintained for 24 h shows an additional peak between 0.54 and 2.51 ms (Figure 7b). This additional peak can be explained by the gel pores connection following activation of the unreacted ash forming a category of intermediate pores. Also, it can be observed that with the increase of the drying time the curves become more flattened and the transition from one category of pores to another is less visible. This phenomenon can be related to the increase of the structure permeability and also to the pores size distribution.

The geopolymers samples with 70% by mass sand of solid components present a higher decrease in the number of gel-type pores (Figure 8b). When the drying time is increased the number of gel-type pores decreases even higher due to the conversion of gel pores in capillary pores and capillary pores in large pores (voids). Therefore, the drying time positively influences the growth of the pores as a result of the reaction between the undissolved ash particles and the activation solution from the gel pores.

However, the lowest pores size distribution was obtained for the samples with 15% ash, 15% glass powder, and 70% sand due to the percentage of compact particles in the analyzed sample volume. Therefore, the coal-ash percentage from the solid component is directly proportional to the matrix volume, its replacement with compact particles results in the decrease of gel pores number (Figure 9b). 

Also, the type of aggregate influences the geopolymers microstructure, by introducing the glass powder into the composition, a decrease of gel pores occurs, but the relative distribution of capillary and large pores is approximately the same. However, when sand is introduced, the gel pores relative distribution decreases, but that of capillary and large pores increases.

**Figure 10:**
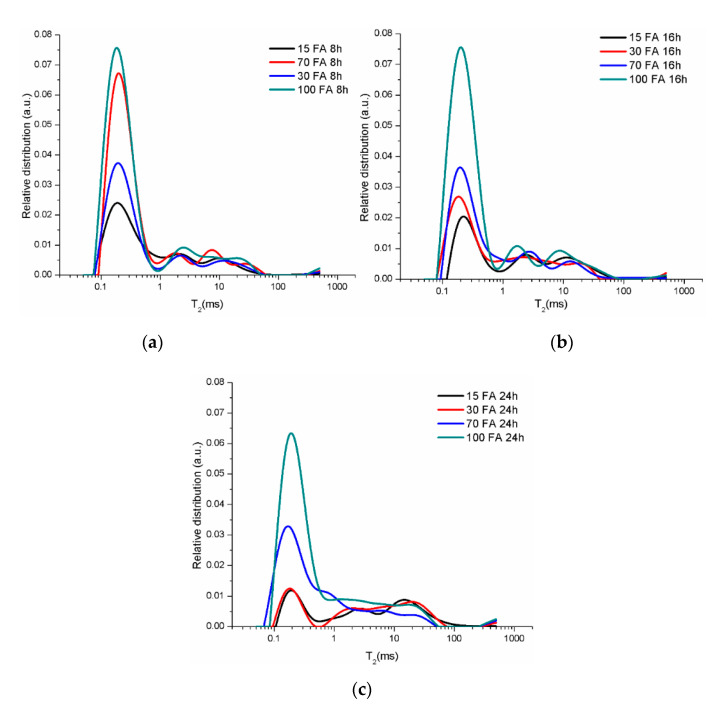
Relative pore size distribution of obtained geopolymers by drying time: (**a**) 8 h; (**b**) 16 h; and (**c**) 24 h.

When the pore size relative distributions are compared depending on the sample composition for 8 h (Figure 10a), 16 h (Figure 10b), and 24 h (Figure 10c) drying time, all the curves present three peaks which area is decreased by the increase of the percentage of the reinforcing particles. The first peak with the smallest area decreases as a result of the coal-ash percentage decreasing. However, the influence of the particles on the relative distribution of capillary and large pores is relatively low. The area values and peaks position (X_1_ and X_2_) on the T_2_ axis are presented in Table 3.

**Figure 11:**
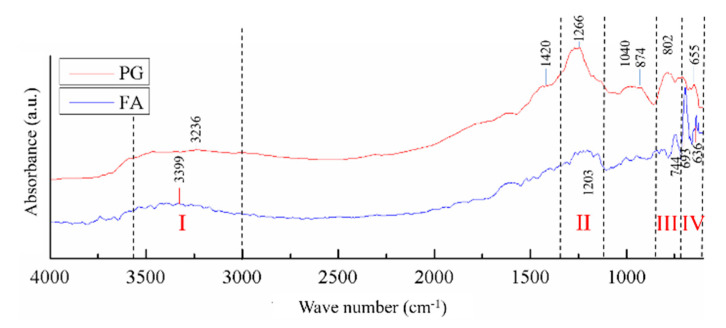
FTIR spectra of FA and PG.

The chemical structure of the obtained geopolymers reveals multiple vibration bands specific to OH^−^ and Si groups (Si-OH), or asymmetric stretching vibrations of the Si-O-Si bridge and Si-O-Al bridge and the stretching vibrations of the Si-O rings. The FTIR spectra (Figure 11) of FA and PG shows a broad signal (I) between 3700 and 3000 cm^−1^ which is attributed to the stretching vibration and bending vibration of OH^−^ groups [38]. The large bandwidth is due to the high degree of hydrogen association with other hydroxyl groups by creating strong links between the OH^−^ and Si (≡Si-OH) groups. The second significant peak (II), between 1150 cm^−1^ and 1250 cm^−^^1^, can be associated with the specific rhythmic band along the covalent bond axis, which is known as stretching vibration of the Si-O-Si groups [39]. The vibration band between 800 and 700 cm^−1^ (III) is specific to the asymmetric stretching vibrations of the Si-O-Al bridge in the compounds [40], (corundum, anorthite) of the analyzed material, and the band between 700 and 600 cm^−1^ is attributed to the Si-O rings [41].

The chemical structure analysis of the raw materials through FTIR confirms the presence, on the analyzed surface, of hydroxide groups and silicon, oxygen and aluminum compounds which correspond to a high percentage of quartz, corundum, anorthite, and vitreous phase.

**Figure 12:**
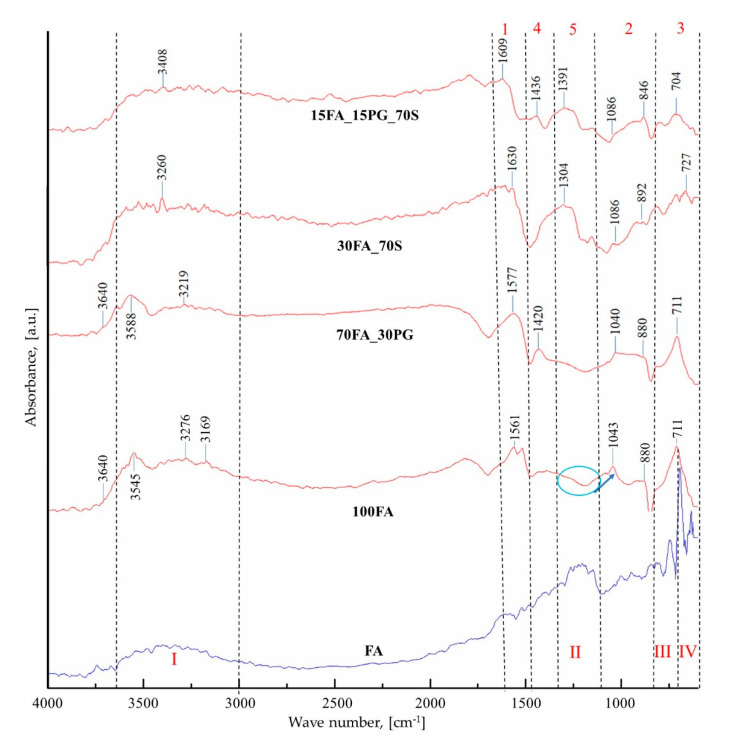
FTIR spectra of raw material (FA) and the analyzed samples.

As a result of the coal-ash activation (Figure 12), the vibration band I show an increase in intensity due to the Si-OH bond’s appearance and OH^−^ group concentration increase. Simultaneously, another vibration band (1) between 1650 and 1480 cm^−1^ appears, which corresponds to the change of the angle between two covalent bonds, known as deformation vibration. In this case, the deformation vibration of the δ-HO-H bonds between the hydrogen and oxygen atoms specific to the adsorbed water molecules [42] is recorded. The bands III and IV undergo significant transformations as a result of the reaction between the compounds rich in aluminum and silicon and the alkaline activator, thus a specific vibration band (3) appears. The position change of the band is attributed to the internal vibrations of the sialates tetrahedra (Si-O-Al, Si-O-Al-O-Si-O, or Si-O-Al-O-Si-O-Si-O) resulting from geopolymerization [24]. Moreover, band II is also shifted to low frequencies (2) as a result of the increase in OH^−^ groups concentration on the analyzed surface and also due to Al^3+^ atoms penetration into the initial Si-O-Si structure forming the N-A-S-H and C-A-S-H phases, a phenomenon specific to zeolites [43]. A high peak corresponds to a high rate of the aluminum atom in the [SiO_4_]^4−^ group penetration, i.e. a higher content of N-A-S-H and C-A-S-H. The main element of influence is the sodium ions concentration in the activator that cause the Si-O bond breakage and increase the ability to incorporate aluminum during the gel phase [44]. This aspect is confirmed by the appearance of the crystalline phase in the structure and the increase of the hygroscopicity of the material by the appearance of the small pores that can be observed in the SEM micrographs (see Figure 4).

Besides the main vibration bands which correspond to silicon, oxygen, aluminum, and hydrogen compounds, another peak at 3640 cm^−1^ which corresponds to OH^−^ stretching vibration appears. Following the replacement of 30% of FA with PG, the FTIR spectrum (Figure 12) does not present significant changes. However, the band I is higher due to the increase of hydroxide groups, introduced in the sample by the activation solutions, to raw material ratio. Moreover, a new band (4) between 1480 and 1370 cm^−1^ appears, which is attributed to the C-O groups in CO_3_^2−^ of calcite, especially, from glass particles [45].

After replacing 70% of coal-ash with sand particles two significant peaks, between 1250 and 1100 cm^−1^, appear on the sample 30FA_70S FTIR spectrum (Figure 12). These peaks correspond to the stretching vibration band of the asymmetric groups of ν(Si–O–Si) and δ(Si–O) [46]. These two groups are characteristic to the quartz from the sand.

The sample 15FA_15PG_70S FTIR spectrum (Figure 12) shows a decrease in the intensity of peaks from the bands 2 and 3 as a result of the aluminum concentration decrease. Therefore, the coal-ash concentration is directly proportional to the number of sialates groups present in the structure.

## 4. Conclusions

The relative pore size distribution of the coal-ash geopolymers confirms the presence of three types of pores in the geopolymers structure. These reveal a high size distribution, ranging from nanometers (gel or capillary pores) to millimeters (large pores or voids). The relative distribution between these three types of pores is influenced by the drying time and also by the percentage of reinforcing particles. Therefore, by increasing the drying time, the gel remaining on the surface of the unreacted or partially dissolved coal-ash particles continues to activate, resulting in the ash spheres opening and pores volume increase. Moreover, by increasing the percentage of the reinforcing particles the number of gel-type pores decreases proportionally. Therefore, the lowest gel-type pores size distribution was obtained for the samples with 15% FA, 15% PG, and 70% Sdue to the percentage of compact particles in the analyzed sample volume. In other words, the coal-ash percentage from the solid component is directly proportional to the matrix volume, its replacement with compact particles results in the decrease of gel pores number. Also, the type of aggregate influences the geopolymers microstructure, by introducing the PG into the composition, a decrease of gel pores occurs, but the relative distribution of capillary and large pores is approximately the same.

FTIR spectroscopy analysis of the obtained geopolymer samples reveals their chemical structure—mainly based, on groups formed between silicon, oxygen, and aluminum atoms, but also hydrogen. As a result of the activation, on the FTIR spectra also appear bands specific to the sialates that confirm the geopolymerization reaction between the raw material and the activator. Moreover, the FTIR spectra of the analyzed samples show a band specific to water molecules that highlights the hygroscopic characteristics of these materials. 

According to this study, the obtained geopolymers contain three types of pores: gel (<50 nm), capillary (50–600 nm) and large pores (pores greater than 600 nm) formed by the arrangement of the OH^−^ and Si groups (Si-OH), Si-O-Si groups, Si-O-Al groups, and Si-O rings.

## Figures and Tables

**Table 1:** Indigenous coal ash oxide chemical composition.

Oxide	SiO_2_	Al_2_O_3_	Fe_x_O_y_	CaO	K_2_O	MgO	TiO_2_	Na_2_O	P_2_O_5_	Oth.^1^
%, weight	47.80	28.60	10.20	6.40	2.40	2.00	1.30	0.60	0.40	0.30

^1^ Sum of chemical elements lower than 0.1%.

**Table 2:** PG oxide chemical composition.

Oxide	SiO_2_	Al_2_O_3_	Fe_x_O_y_	CaO	Na_2_O	MgO	Oth.^1^
%, weight	71.69	1.81	0.93	13.2	9.89	2.40	0.08

^1^ Sum of chemical elements lower than 0.1%.

**Table 3:** Peaks areas and positions on the T_2_ axis.

Sample	Drying Time	Peak 1			Peak 2			Peak 3		
		X1 (ms)	X2 (ms)	Area (a.u.)	X1 (ms)	X2 (ms)	Area (a.u.)	X1 (ms)	X2 (ms)	Area (a.u.)
15FA	8 h	0.07	1.07	0.01	1.07	5.17	0.02	5.17	86.81	0.11
30FA	8 h	0.07	0.91	0.01	0.91	5.24	0.01	5.24	61.16	0.12
70FA	8 h	0.09	0.89	0.01	0.89	3.71	0.01	3.71	63.17	0.18
100FA	8 h	0.07	0.91	0.02	0.91	5.63	0.03	5.62	50.40	0.17
15FA	16 h	0.11	0.88	0.01	0.88	5.08	0.02	5.08	76.65	0.20
30FA	16 h	0.08	0.69	0.01	0.69	9.91	0.05	9.91	40.40	0.10
70FA	16 h	0.09	1.25	0.01	1.25	6.42	0.03	6.42	60.60	0.12
100FA	16 h	0.07	0.80	0.02	0.80	3.83	0.02	3.83	91.74	0.24
15FA	24 h	0.10	0.53	0.01	0.53	4.94	0.02	4.94	150.2	0.35
30FA	24 h	0.09	0.56	0.01	0.56	5.57	0.02	5.57	76.65	0.30
70FA	24 h	0.06	1.65	0.01	1.65	12.65	0.05	12.65	53.02	0.09
100FA	24 h	0.08	0.82	0.01	0.82	10.37	0.07	40.37	55.78	0.18
